# Investigating the teacher’s perceptions of classroom management and teaching self-efficacy during Covid-19 pandemic in the online EFL courses

**DOI:** 10.1186/s40862-022-00152-7

**Published:** 2022-10-10

**Authors:** Zahra Akbarzade Farkhani, Ghazal Badiei, Farzad Rostami

**Affiliations:** 1 Islamic Azad University, Quchan Branch, Quchan, Iran; 2 Islamic Azad University, West Tehran Branch, Tehran, Iran; 3 Islamic Azad University, Baneh Branch, Baneh, Iran

**Keywords:** Classroom management, COVID-19, EFL teachers, Online courses, Synchronous-based online learning

## Abstract

During the coronavirus pandemic, online education continued to expand across varied educational factors. Therefore, the teachers had to develop and change some of the strategies used in their classes previously. Online classroom management is a synchronous-based online learning environment in education that is worthwhile to modify. For this purpose, the current study sought to understand the perceptions of classroom management and teaching self-efficacy by Iranian EFL teachers during the Covid-19 pandemic. Concerning sampling, 100 male and female English teachers constituted the study sample. Data were collected via Online Teaching Self–Efficacy Inventory questionnaire through different online platforms. The findings reflected that EFL teachers could select appropriate classroom management during online and face-to-face classes. In addition, the teachers had a positive attitude toward managing the classroom during the Covid-19 pandemic. The implications of this study may open up new perspectives into successful pedagogy for, teachers and students in outbreak days.

## Introduction

Classroom management is one of the most critical issues in educational settings (Yilmaz & Cavas, 2008) and vital in constructing effective learning environments (Akar et al., 2010). The emergence of the Covid-19 pandemic made countries change their instructional system. Traditional face-to-face teaching, that was using for many years, was replaced by entirely online e-learning courses. In the meanwhile, the management of the online courses changed, and new strategies were adopted. Several studies supported that classroom management has correlations with some variables including self-control, responsibility, psychological well-being, and discipline, influencing academic outcomes (Bean, 2007; Brophy, 1988; Fareh, 2018; Jones & Jones, 2004; Marzano, Marzano, & Pickering, 2003; Savage & Savage, 2009; Wang et al., 1993). Moreover, evidence suggests that teachers with professional classroom skills impact learners’ behaviors positively (Emmer & Emertson, 2013; Fareh, 2018; Raider-Roth, 2005; Walker, Ramsey, & Gresham, 2004).

Although many studies have been carried out about classroom management, few papers directly investigate the potential role of technology in online classes (Cho et al., 2020). In an online educational setting, recently teachers have been able to practice classroom management in computer-simulated classes- rooms (Judge, Bobzien; Maydosz, Gear, & Katsioloudis, 2013) or connect with classroom management coaches online (Rock et al., 2013). However, the investigation of teachers’ insights of classroom management during online classes has been less exported particularly in EFL Iranian context. Acquiring such information will provide a deeper understanding of challenges related to classroom management and online courses to find an advanced solution and improve a well-adjusted educational system that is forced to be integrated with technology. Hence, the current research addresses the following research questions.

1. What are the EFL teachers' views on online classroom management?
2. How confident do EFL teachers feel in preparing, conducting, and managing online courses?
3. What are teachers’ perceptions toward the kind of applications used during online courses?

## Review of literature

One of the key factors of professional teacher competence, and effective learning in face-to-face or online classrooms is classroom management. There are multiple definitions of classroom management. Brophy (1996) introduced it as “actions taken to create and maintain a learning environment conducive to successful instruction” (p. 5). In addition, Marzano (2003) proposed “establishing and reinforcing rules and procedures, carrying out disciplinary actions, maintaining effective teacher and student relationships, and maintaining an appropriate mental set for management” (p. 88). Later Weber et al. (2018) noted crucial variables underlying classroom management including monitoring, which refers to keeping teachers’ awareness of events continuously that may happen in the classroom (Gold & Holodynski, 2017; Kounin, 1970; Wolff, 2015). For example, feedback, appreciation, and prompt responses to misbehaviors are included in this component (Doyle, 2006; Evertson & Emmer, 2013; Little & Akin-Little, 2008).

Another variable is how to manage momentum (Thiel et al., 2012). It refers to making a balance between little wasted time and activities in the class (Pianta et al., 2012). Also, it contains clarifying the instructions, fulfilling the purpose and the structure of the lesson, appropriate materials, providing group focus and classroom conditions (Doyle, 2006; Kounin, 1970). Another significant aspect of classroom management is establishing rules and regulations which can positively affect learners' behavior (Emmer & Emertson, 2013; Fareh, 2018; Raider-Roth, 2005; Walker, Ramsey, & Gresham, 2004). In 2004, Jones and Jones published a paper about classroom management in the context of three approaches entail *counseling* which focuses on maintaining learners under discipline, *behaviorist approach* which concentrates on modification techniques for learner’s undesirable behaviors, and *preventive approach* which emphasizes to hinder learners’ misbehaviors.

A large and growing body of literature has investigated the importance of classroom management. In this regard, Kounin, (1970) reported this factor increases learning, reduces interpretation, and maintains an influential environment. A positive correlation was identified between teachers ‘classroom management and learners’ achievement (Hattie, 2009) and academic optimism (Murray & Zvoch, 2011). This component creates an effective learning environment (Fareh, 2018) influences teachers’ mental health, and can keep them from burnout and stress (Friedman, 2006; López et al., 2008). Moreover, the role of teachers’ experience in providing effective classroom management has been investigated in different studies to emphasize the distinction between knowledge and vision of the classroom among experts and novice practitioners (Gold & Holodynski, 2017; Wolff et al., 2017). In this field of study, one of the scales of classroom management is the Behavior and Instructional Management Scale developed and validated by (Martin & Sass, 2010). Behavior management is associated with teachers’ attempts to prevent and respond to learners’ misbehavior, but instructional management comprises goals, plans, rules, that teachers apply to provide instructions in a class to engage learners.

### Classroom management in EFL context

The evidence presented in literature manifested that “classroom management as inherently equal to all subject matter areas and so ignored the distinctive characteristics of classroom management for specific content areas” (Macías, 2018, p. 155), whereas he believed that according to eleven language teachers’ characteristics introduced by Borg (2006), three dramatic factors including the use of target language, patterns of interaction, and communicative competence influence classroom management in EFL context. Macías (2018) elaborated that, In the EFL context, teachers should apply medium language to give them instruction when students might not understand by the term of interaction patterns or group work, which might not be essential in other subjects.

Different studies exist in the literature regarding classroom management. Lee and van Vlack’s (2018) research on 127 English south Korean teachers showed that “Enjoyment and, surprisingly, anger also correlated positively with classroom management self-efficacy, while frustration correlated negatively. This shows a significant relationship between teachers’ emotions and classroom management self-efficacy” (p. 12). They suggested that future studies could be done as longitudinal research or on a larger sample. Akman (2020) examined 608 secondary school students in Turkey and found that “classroom management was an important element influential in students’ perceptions of confidence and stress” (p. 341). The suggestions for future studies were conducting mixed-methods research and considering a larger sample. Egeberg et al.'s (2021) mixed-methods research investigated 50 Australian teachers’’ perceptions about classroom management. “Effective classroom management is multidimensional including caring relationships, high expectations, and opportunities for engagement, participation, and contribution (p. 121). It was not in the EFL context. Recently Traditional face-to-face teaching has been shifted into online courses. It is required to update teaching strategies in a virtual classroom context.

### Online classroom

The characteristics of an online classroom identified by Lathifah et al. (2020) are as follows: (a) the class session must be in real-time connecting the teacher and the students synchronously, (b) the teacher and the students are distinguished by location, and (c) the class uses a platform closed for certain people (p. 264). Although virtual class lacks physical contact between teachers and learners and managing the rules is demanding, this sort of class is more flexible to attend and learner-based (Rufai et al., 2015). The term online classroom refers to the whole teaching–learning procedures carried out in online ways. According to Taghizadeh and Amirkhani (2022), online teaching includes planning, organizing, leading, controlling, and administering the online materials in the classroom; online courses can be as successful as face-to-face experiences with effective teachers' management. Ghateolbahra and Samimi, (2021) mentioned that "the professional development of online education, especially in the field of classroom management, requires a set of practical strategies, knowing how to communicate well with students, having an effective classroom management program, and managing asynchronous discussions and online teamwork" (p. 510). The study suggested that more research should focus on teachers' knowledge and skills in effective classroom management in other disciplines and at different levels.

Durak and Saritepeci’s (2017) mixed-method study, among 52 teachers as participants, found that technology use positively influenced classroom management. According to the results of these studies, teachers emphasized the level of their teacher's IT literacy as the most significant element of classroom management in technology-assisted courses. Durak and Saritepeci (2017) added although it seems that younger teachers could be better at using technology in classroom management, the result of their research was vice versa. The critical factor was that older teachers with higher experience had fewer problems managing their classrooms. Most of the previous studies related to classroom management were from the general perspective of teaching, and the investigation of the role of this factor in foreign language teaching context is missing and less explored (Macías, 2018). Although the vast amount of classroom management research was based on face-to-face classes, a systematic review conducted by (Cho et al., 2020) suggested that “there is a pressing need for scholars and practitioners to view the landscape of possibilities when it comes to classroom management and technological advancement” (p. 2). Evidence shows it is a need to investigate how teachers view the management of online classes in the EFL context that the current paper tries to cover this gap through a quantitative study.

## Methodology

### Participants

It was difficult to find enough participants during the coronavirus pandemic. The survey was performed online and sent to 377 EFL teachers through some web-based platforms (Telegram 36%, WhatsApp 57%, and others about 7%). Among 339 teachers receiving the survey, only 100 answered the questionnaire. Among the participants completing the study, there were 65% females and 35% males, with an average allocated time of eleven minutes. The teaching experience ranged from novice researchers to those with more years of experience. All of the participants attending this study came from Iran, of whom 44.4% were teaching at intermediate to upper-intermediate levels of private language institutions, 45.5% at public education, and about 10% didn't mention their teaching place. Most of them held an MA or BA degree in different branches of English studies, including English literature, English teaching, English translation, and some teachers were Ph.D. candidates in English teaching. All of them had about three semesters teaching online. For non-probability sampling, the participants were selected based on convenience sampling.

### Instrument

The classroom management subcategory was extracted from Online Teaching Self-Efficiency Inventory (OTSEI) to answer the preceding research questions and collect the data from participants. The questionnaire is based on the work of Dr. Kevin P. Gosselin in Australia (Gosselin, 2009). It was the primary research questionnaire (See Appendix A). OTSEI is a Likert scale survey consisting of 46 items to assess online teaching, management, and efficacy of the teacher (Maddux & Gosselin, 2012). This questionnaire was used because classroom management is one of its categories. According to that, it was adopted and only nineteen related items were used in this study. These items examined teachers’ competence for online teaching, including how confident EFL teachers felt in preparing, conducting, assessing, and aligning online courses and whether they evaluated the learners in online classes, checked assignment, provided feedback, and set the learning goals in the online classroom. Also, there is a lack of domain-specific research instruments for measuring the online classroom management of EFL teachers. Data collected from OTSEI across each stage provided the necessary information for this quantitative research. Some online school teachers were supposed to answer the scale to indicate how confident they were in accomplishing the activities by selecting a number for each question on a scale ranging from 0 (No confidence) to 10 (Complete confidence). It is noteworthy that the questionnaire also included some subcategories. The sections include selection of technological resources, virtual interaction, unit content migration (the ability to successfully transfer instructional materials from face-to-face to online units), online courses alignment, online resources, and web-based unit structure (the ability to construct and design an online team including a clear organizational structure and facilitating software and communication guidelines). In addition to the information gathered in the survey, some demographic information such as the age of participants, gender, ethnic identity, current teaching position, years of experience, and online teaching experience was also collected (see Appendix A). Alpha reliabilities of the ranking scales ranged from 0.84 to 0.95, reflecting suitable internal consistency. The average variance accounted for the five single-factor scales ranged from 45.93 to 64.38%, with an average of 53.16% of explained variance, providing evidence for good factor validity (Stevens, 1996).

### Procedure and data analysis

This study was carried out among English teachers from different cities in Iran. The EFL teachers received the English version of the OTSEI questionnaire through a web-based platform. To keep themselves healthy and away from infection to Coronaviruses, the participants were reluctant to be interviewed face to face or observed. Thus, the questionnaire was the best choice to be sent out to the participants online and sent back to the authors. Although filling in the questionnaire was taking about ten minutes, some participants delivered the questionnaire late, about a week to ten days, because of different problems such as not having enough time or engaging in online classes. They answered the questionnaire anonymously, but each participant left an email address in their answer sheet for any other request in case of need. They sent a word document of the survey that they filled out for the authors.

When the schools closed their doors to face-to-face instruction, English language teachers had to manage their classrooms via online courses. Therefore, all the subjects had online teaching experience. After data collection, this questionnaire was addressed by calculating means and standard deviations of the teacher classroom management in online teaching courses through OTSEI survey scores for the five measures, including the selection of online resources, virtual interaction, and units content migration, online course alignment, and web-based unit structure. Descriptive statistics were applied to describe the collected data. Finally, SPSS software was used for analyses due to the normality of data.

## Results

### Examining the first research question

The first research question of the current study aimed to investigate how EFL teachers viewed online classroom management. It means whether they could manage their online classes similar to face to face ones or they could adopt a teaching style that allowed for the facilitation of learning through guidance.

Table 1 displays the participants’ numbers and the respective percentages of responses for each questionnaire item related to online classroom management. Every item begins with (in the context of online units, I could …) for example, in the context of online units, I could get students to work together in my classes. The items are arranged on a scale ranging from 0 (No confidence) to 10 (Complete confidence).

**Table 1:** The participants’ numbers and the respective percentages of responses for each item of the questionnaire related to online classroom management

19	Get students to work together in my classes	0 0%	3 3%	3 3%	3 3%	8 8.1%	10 10.1%	12 12.1%	14 14.1%	11 11.1%	12 12.1%	23 23.2%
20	Overcome the influence of adverse student interactions	1 1%	2 2%	3 3%	2 2%	5 5.1%	10 10.1%	12 12.1%	20 20.2%	22 22.2%	10 10.1%	12 12.1%
21	Encourage my students to ask questions	0 0%	0 0%	3 3.1%	1 1%	5 5.1%	4 4.1%	9 9.2%	14 14.3%	19 19.4%	14 14.3%	29 29.6%
22	Promote student participation in my units	0 0%	0 0%	2 2%	1 1%	3 3.1%	6 6.1%	9 9.2%	7 7.1%	26 26.5%	18 18.4%	26 26.5%
23	Project a positive virtual social presence (the perception of being real)	0 0%	0 0%	1 1%	5 5.1%	4 4.1%	14 14.3%	16 16.3%	12 12.2%	15 15.3%	12 12.2%	19 19.4%
24	Effectively express emotion within the online environment	1 1%	0 0%	1 1%	4 4%	3 3%	12 12.1%	11 11.1%	14 14.1	23 23.2%	10 10.1%	20 20.2%
25	Use emotion to effectively enrich communication	0 0%	0 0%	1 1%	1 1%	5 5.1%	10 10.2%	8 8.2%	14 14.3%	21 21.4%	15 15.3%	23 23.5%
26	Adopt a teaching style that allows for the facilitation of learning through guidance	0 0%	0 0%	1 1%	1 1%	3 3.1%	14 14.3%	6 6.1%	26 26.5%	21 21.4%	8 8.2%	18 18.4%
27	Manage the pace of facilitating interaction	0 0%	1 1%	0 0%	0 0%	3 3.1%	11 11.2%	15 15.3%	17 17.3%	25 25.5%	12 12.2%	14 14.3%
28	Adequately convey that I am available for consultation	1 1%	0 0%	0 0%	0 0%	4 4%	6 6.1%	10 10.1%	17 17.2%	17 17.2%	21 21.2%	23 23.2%

0 = no confidence, 10 = complete confidence

As indicated in Table 1, the number of participants who responded to the items with an inclination towards the complete confidence end was substantially higher than those who responded to the items with a tendency towards the no-confidence. For instance, scrutiny of item 19 indicates that only 17 (17.3%) of the responses belonged to 0 while 82 (82.7%) of the answers belonged to the 10. Other items in the above table follow the similar pattern, and few participants checked 0 as no-confidence scale. Thus, it can be concluded that EFL teachers were confident in their online classroom management.

### Examining the second research question

The second research question of the present study explored how confident EFL teachers felt in preparing, conducting, assessing, and aligning online courses. Whether or not they evaluate the learners in online classes, if they check assignment, get feedback, get the learning goals in the online classroom. Table 2 shows the participants’ numbers and the respective percentages of responses for each questionnaire item related to preparing, conducting, assessing, and aligning online courses.

**Table 2:** The participants’ numbers and the respective percentages of responses for each item of the questionnaire related to preparing, conducting, assessing, and aligning online courses

In the context of online units, I could …		0 = no confidence					10 = complete confidence					
36	Evaluate the degree to which my unit learning outcomes have been met	0 0%	3 3.1%	3 3.1%	2 2%	4 4.1%	7 7.1%	15 15.3%	16 16.3%	15 15.3%	17 17.3%	16 16.3%
37	Use strategies to increase my students’ memory of my unit content	0 0%	0 0%	2 2.1%	2 2.1%	5 5.2%	11 11.3%	12 12.4%	15 15.5%	24 24.7%	12 12.4%	14 14.4%
38	Provide my students with detailed feedback about their academic progress	0 0%	0 0%	0 0%	2 2%	7 7.1%	8 8.2%	13 13.3%	19 19.4%	22 22.4%	9 9.2%	18 18.4%
39	Determine the most appropriate evaluation method for a particular unit	0 0%	0 0%	3 3.1%	1 1%	6 6.2%	7 7.2%	18 18.6%	17 17.5%	22 22.7%	10 10.3%	13 13.4%
40	Clearly articulate the learning goals that I expect my students to attain	0 0%	0 0%	1 1%	0 0%	4 4.1%	9 9.2%	15 15.3%	17 17.3%	25 25.5%	12 12.2%	15 15.3%
41	Connect unit assignments with the stated learning outcomes	0 0%	0 0%	0 0%	0 0%	4 4.1%	9 9.3%	12 12.4%	21 21.6%	21 21.6%	17 17.5%	13 13.4%
42	Accurately assess the depth of students’ learning	0 0%	1 1%	1 1%	2 2%	6 6.1%	13 13.3%	10 10.2%	13 13.3%	24 24.5%	12 12.2%	16 16.3%
43	Accurately assess the depth of students’ level of engagement	0 0%	2 2.1%	1 1%	2 2.1%	4 4.1%	14 14.4%	20 20.6%	13 13.4%	18 18.6%	12 12.4%	11 11.3%
44	Engage students from a variety of cultural backgrounds	3 3.1%	1 1%	0 0%	2 2%	3 3.1%	11 11.2%	12 12.2%	16 16.3%	20 20.4%	11 11.2%	19 19.4%
45	Engage students who have a wide variety of familiarity with online learning	2 2%	1 1%	1 1%	1 1%	0 0%	8 8.2%	15 15.3%	16 16.3%	20 20.4%	17 17.3%	17 17.3%
46	Use written instructions to facilitate student engagement in online units	1 1%	0 0%	1 1%	1 1%	4 4.1%	12 12.2%	11 11.2%	18 18.4%	18 18.4%	16 16.3%	16 16.3%

0 = no confidence, 10 = complete confidence

As presented in Table 2, the number of participants who responded to the items with an inclination towards the complete confidence end was considerably higher than those who answered the items with a tendency towards 0. For example, a look at item 36 shows that only 19 (19.1%) of the responses belonged to 0, while 81 (82.9%) of the answers belonged to10. Other items in the above table follow a similar pattern. Thus, it can be concluded that EFL teachers in the current study were confident in preparing, conducting, assessing, and aligning online courses so that the online classroom could not affect the quality of classroom management and applying interaction strategies.

### Examining the third research question

The third research question of this study explored teachers’ perceptions toward the kind of applications used during online courses and selecting the best one for their teaching. Also this question explored the way the teachers learned to use new technologies in their units. Table 3 depicts the participants’ numbers and the respective percentages of responses for each item of the questionnaire related to teachers’ perceptions toward the kind of applications used during online courses.

**Table 3:** The participants’ numbers and the respective percentages of responses for each item of the questionnaire related to teachers’ perceptions toward the kind of applications used during online courses

In the context of online units, I could …		0 = no confidence					10 = complete confidence					
11	Select the appropriate software applications to use for my classes	2 2%	1 1%	7 7.1%	5 5%1	1 1%	12 12.1%	12 12.1%	10 10.1%	12 12.1%	13 13.1%	24 24.2%
12	Obtain the appropriate copyright permissions	10 10.3%	3 3.1%	10 10.3%	4 4.1%	10 10.3%	8 8.2%	9 9.3%	12 12.4%	7 7.4%	12 12.4%	12 12.4%
13	Discern between technological applications that require different levels of bandwidth	6 6.1%	2 2%	2 2%	6 6.1%	12 12.2%	16 16.3%	7 7.1%	13 13.3%	16 16.3%	8 8.2%	10 10.2%
14	Determine how difficult various types of technology will be for my students to use	3 3%	1 1%	1 1%	2 2%	8 8.1%	10 10.1%	16 16.1%	23 23.2%	13 13.1%	11 11.1%	11 11.1%
15	Select the online unit technology that is most efficient for the delivery of materials to students	2 2.1%	1 1%	2 2.1%	4 4.2%	11 11.5%	12 12.5%	11 11.5%	15 15.6%	12 12.5%	10 10.4%	16 16.7%
16	Learn how to use new technologies in my unit without support from my institution	1 1%	3 3%	4 4%	4 4%	2 2%	10 10.1%	10 10.1%	13 13.1%	21 21.2%	13 13.1%	18 18.2%
17	Select the unit technology compatible with students’ networks and platforms	0 0%	0 0%	5 5.2%	3 3.1%	6 6.2%	17 17.5%	13 13.4%	13 13.4%	19 19.6%	10 10.3%	11 11.3%
18	Manage the time requirements needed for learning unit technology	0 0%	1 1%	1 1%	3 3.1%	7 7.1%	6 6.1%	9 9.2%	21 21.4%	22 22.4%	11 11.2%	17 17.33%

0 = no confidence, 10 = complete confidence

As seen in Table 3, the number of participants who responded to the items with an inclination towards 10 was considerably higher than those who answered the items with a tendency towards 0. For instance, in item 11, only 16 (16.1%) of the responses belonged to the no-confidence end, while 83 (83.83%) of the answers belonged to the complete confidence end. Other items in the above table have a similar pattern. Thus, EFL teachers showed confidence in using the kind of applications in online courses. The results reflect that even though the teachers have difficulty with digital literacy and working with different platform in contraction with learners, they mediated various applications well to better control the class and qualify the online courses as they performed in face to face classes.

## Discussion

The present study sought to understand EFL teachers' perceptions towards classroom management during the Covid-19 pandemic. The first research question of this study concerned EFL teachers’ views on online classroom management. The results suggest that EFL teachers were confident in developing online courses and managing the online environment and instructions. They could adopt some teaching styles that allowed for the facilitation of learning through their guidance and conveyed their face-to-face class management strategies to online courses. This result confirms the finding of Diamond Hicks (2012), who explored the correlation between classroom management and some other influencing factors such as self-efficiency through the same questionnaire which authors applied in the current research paper. Moreover, the results of the present investigation are in accord with a recent qualitative study that performed by (Rufai, Alebious & Adeakin, 2015). The current paper indicated that although virtual classes lack physical interaction between learners and teachers, managing this sort of class is more flexible to attend. It can be also learner-based such as the typical situations of teaching in which EFL teachers are capable of establishing a positive social climate that engages students in learning via an online course. The other objective of this study was to explore how confident EFL teachers felt in preparing, conducting, assessing, and aligning online courses. In other words, this question aimed to see whether the teachers could effectively align learning objectives, unit assignments and learning activities, assessment strategies, and procedures with online courses. Examining the ten items of the OTSEI questionnaire exclusively concerned with this question showed that EFL teachers were confident in preparing, conducting, assessing, and aligning online courses. This means that in an online course, the EFL teachers can indicate their ability to manage the learning environment by providing whatever is necessary to have good teaching. On the other hand, when the online course alignment by the teacher is suitable, as Kirtman (2009) mentioned, there are similar learning outcomes whether the teacher teaches in a traditional or online class. In consistent with the findings of Francis and Oluwatoyin (2019), the results showed that online classroom management was easier and the class atomospher was cooler when teachers improved their various technological knowledge.

The last research question explored teachers’ perception toward the kind of applications used during online courses for managing the online classroom. In the context of online teaching, it is necessary to select appropriate technological resources. In other words, the teachers must have the ability to choose, utilize, and determine the appropriateness of technology for managing the classroom well in online courses to enhance the students' learning and enrich instruction. This study concluded that EFL teachers could select the appropriate software application to use in their classes to deliver materials to the students and develop their instruction despite the problems like internet speed and weak digital platforms that Iranian teachers have been encountered. This outcome agrees with a study (Bates and Poole, 2003), indicating that effective teaching enables the teachers to look at the use of technology to improve teaching and learning in complementary ways. As a result, they can make the right choices of using media and technology to enhance education significantly. In the same line, Graham et al. (2020) and Taghizadeh and Amirkhani (2022) conducted a research by means of questionnaire. They mentioned that teachers might view technology integration as beneficial when it increases productivity and social influence and fosters pedagogical changes. However, the results indicated the learners and teachers are required to improve their digital literacy and behavior to facilitate the management of online classes.

## Conclusion

This study was an attempt to contribute to the effect of online teaching on classroom management by EFL teachers during the time that the way of teaching changed because of the coronavirus pandemic. The findings of the current study illustrated the importance of online courses and managing the classroom while showing that EFL teachers could select appropriate materials for teaching English and manage their classes even in the online teaching context. Furthermore, it showed that EFL teachers were confident in preparing, conducting, assessing, and aligning online courses. They could use technology and suitable software applications to develop their instruction and manage their online classes as they did in their face-to-face courses. In addition, there was no difference between male and female teachers in this procedure. It can be a good opportunity for teachers to improve their technical skills to use in their next face-to-face classes in the future. Thus, most teachers have no problems with managing the classroom in online courses.

One of the implications of the study can be for instructors and educators who may think to design the factors of online management classroom and other dimensions of education. The results of the present study contribute to directing teachers to consider potential strategies that fit online classes. In addition, it can help educators and other researchers to continue exploring the way technology and online platform affect how teachers can act in online classes and rethink some strategies that can help them to enrich their teaching and student learning. Moreover, according to the findings of this research and other similar projects, policy and curriculum makers must consider that there should be a review of teaching and its methods in the post-Corona era and some online teacher training courses can be held for teachers to be able to manage their online courses more efficiently because it seems students and teachers are interested in online education, although there are some problems in this area. One of the limitations of this study was utilizing one instrument to gather quantitative data. However, researchers could obtain more profound results by considering qualitative data. The same research can be carried out via a broader population, with more participants from different parts of the world. Moreover, future studies can use some other instruments such as observations, surveys, and other data analysis methods. The impacts of other variables such as age, gender, socio-economic status, technology importance, and distress of teachers were not studied in this research and need further investigation.

## Data Availability

The data will be available upon request.
